# Corrigendum: Patterns of within-host spread of *Chlamydia trachomatis* between vagina, endocervix and rectum revealed by comparative genomic analysis

**DOI:** 10.3389/fmicb.2024.1441327

**Published:** 2024-06-25

**Authors:** Sandeep J. Joseph, Sankhya Bommana, Noa Ziklo, Mike Kama, Deborah Dean, Timothy D. Read

**Affiliations:** ^1^Division of STD Prevention, Centers for Disease Control and Prevention, Atlanta, GA, United States; ^2^Department of Pediatrics, University of California, San Francisco, Oakland, CA, United States; ^3^Ministry of Health and Medical Services, Suva, Fiji; ^4^Department of Medicine, University of California, San Francisco, San Francisco, CA, United States; ^5^Department of Bioengineering, Joint Graduate Program, University of California, San Francisco, San Francisco, CA, United States; ^6^Department of Bioengineering, Joint Graduate Program, University of California, Berkeley, Berkeley, CA, United States; ^7^Bixby Center for Global Reproductive Health, University of California, San Francisco, San Francisco, CA, United States; ^8^Benioff Center for Microbiome Medicine, University of California, San Francisco, San Francisco, CA, United States; ^9^Division of Infectious Diseases, Department of Medicine, Emory University School of Medicine, Atlanta, GA, United States

**Keywords:** *Chlamydia trachomatis*, single nucleotide polymorphisms, single variable polymorphisms, sexually transmitted diseases, chlamydiae

In the published article, there was an error in Figure 2 as published. Some of the colors in the “ompA genotype” column of the heatmap did not match the true genotype of strain in the phylogenetic tree. The corrected Figure 2 and its caption appear below.

**Figure 2:**
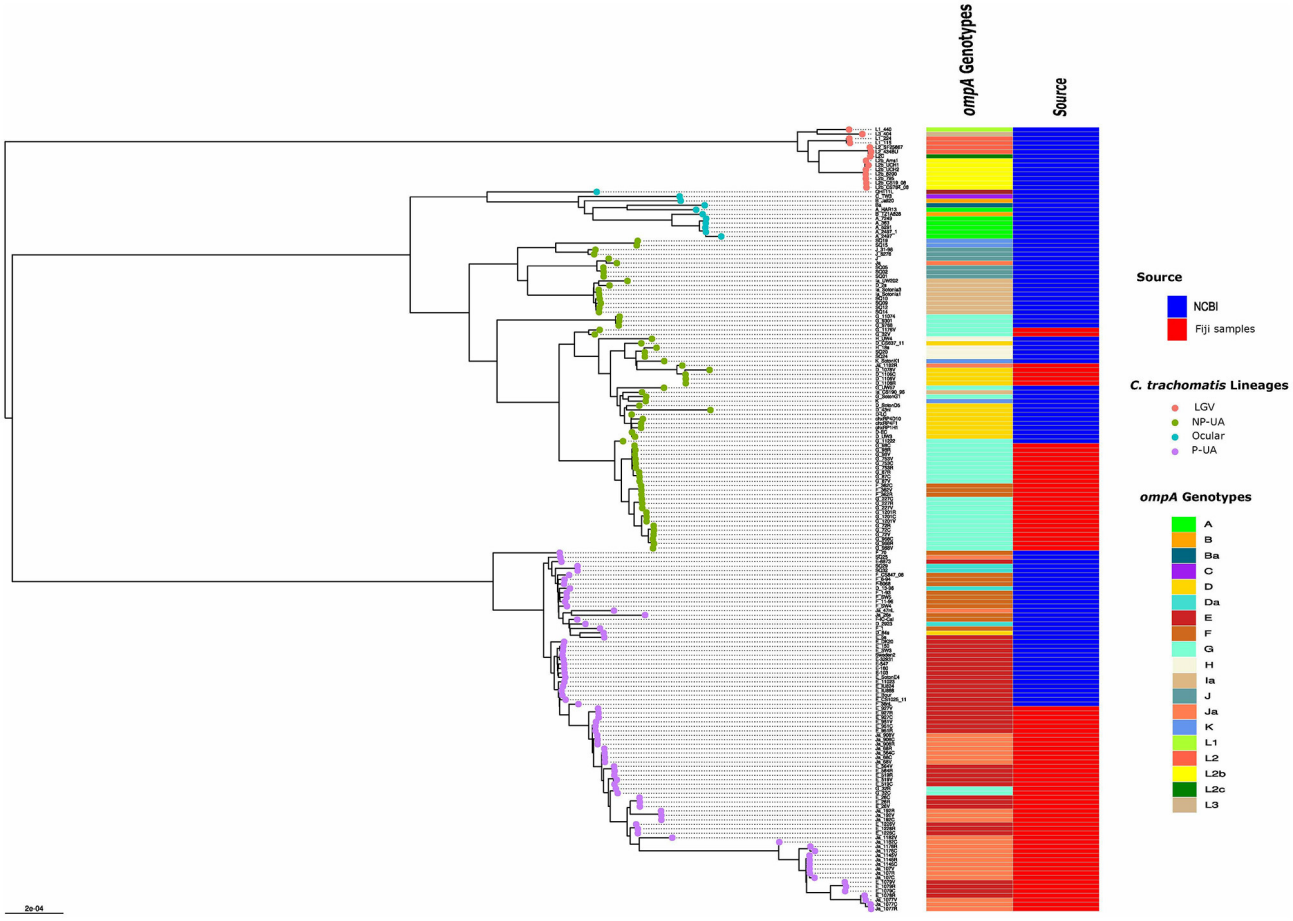
Global phylogeny with clade designations. The global phylogeny of high-quality *C. trachomatis* Fiji genomes plus selected complete *C. trachomatis* reference and clinical genomes representing global diversity from the National Center for Biotechnology Information (NCBI). Sample names are < *omp*A genotype > - < participant ID > - < body site code, where C = endocervix, R = rectum and V = vagina >. The round tips are colored by the 4 clade designations [LGV, ocular, prevalent- urogenital and anorectal (P-UA), non-prevalent urogenital and anorectal (NP-UA)]. The first column to the right of the tree denotes the *omp*A genotype with code at the lower right; the second column represents the source of the genomes from NCBI or the Fijian samples.

The authors apologize for this error and state that this does not change the scientific conclusions of the article in any way. The original article has been updated

